# Liver Metastasis in Cancer: Molecular Mechanisms and Management

**DOI:** 10.1002/mco2.70119

**Published:** 2025-02-27

**Authors:** Wenchao Xu, Jia Xu, Jianzhou Liu, Nanzhou Wang, Li Zhou, Junchao Guo

**Affiliations:** ^1^ Department of General Surgery Peking Union Medical College Hospital Chinese Academy of Medical Sciences and Peking Union Medical College Beijing China; ^2^ Key Laboratory of Research in Pancreatic Tumor Chinese Academy of Medical Sciences Beijing China; ^3^ National Infrastructures for Translational Medicine Peking Union Medical College Hospital Beijing China; ^4^ State Key Laboratory of Complex Severe, and Rare Diseases Peking Union Medical College Hospital Chinese Academy of Medical Sciences and Peking Union Medical College Beijing China; ^5^ State Key Laboratory of Fine Chemicals Department of Pharmaceutical Sciences School of Chemical Engineering Dalian University of Technology Dalian China; ^6^ Department of Colorectal Surgery State Key Laboratory of Oncology in South China Sun Yat‐sen University Cancer Center Guangdong Provincial Clinical Research Center for Cancer Guangzhou China

**Keywords:** liver metastasis, spatial oncology, therapeutics, tumor microenvironment

## Abstract

Liver metastasis is a leading cause of mortality from malignant tumors and significantly impairs the efficacy of therapeutic interventions. In recent years, both preclinical and clinical research have made significant progress in understanding the molecular mechanisms and therapeutic strategies of liver metastasis. Metastatic tumor cells from different primary sites undergo highly similar biological processes, ultimately achieving ectopic colonization and growth in the liver. In this review, we begin by introducing the inherent metastatic‐friendly features of the liver. We then explore the panorama of liver metastasis and conclude the three continuous, yet distinct phases based on the liver's response to metastasis. This includes metastatic sensing stage, metastatic stress stage, and metastasis support stage. We discuss the intricate interactions between metastatic tumor cells and various resident and recruited cells. In addition, we emphasize the critical role of spatial remodeling of immune cells in liver metastasis. Finally, we review the recent advancements and the challenges faced in the clinical management of liver metastasis. Future precise antimetastatic treatments should fully consider individual heterogeneity and implement different targeted interventions based on stages of liver metastasis.

## Graphical abstract

This review focuses on the molecular mechanisms and clinical management of tumor liver metastasis. Liver metastasis is a primary manifestation of the aggressive dissemination of several extrahepatic tumors. Many risk factors, such as individual genetic background, liver diseases, diet, trauma, and medication, may accelerate this process. In terms of temporal trajectory, liver metastasis can be mainly divided into three continuous yet distinct phases, accompanied by the functional differentiation and spatial remodeling of resident and recruited cells. Achieving effective control and even cure of liver metastasis by removing tumors or reducing the tumor burden is the ultimate goal of current preclinical animal experiments and clinical explorations (“turning back the clock”). Despite many challenges, comprehensive clinical management of patients with liver metastasis under multidisciplinary treatment models remains a promising option.2

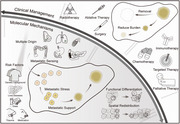

## 1 Introduction

A tumor is a localized tissue proliferation characterized by uncontrolled growth and aggressive dissemination [1]. Liver metastasis is a primary manifestation of the aggressive dissemination of several extrahepatic tumors [2, 3]. Notably, the incidence of liver metastatic tumors is nearly 40 times higher than that of primary liver tumors [4]. When metastasis occurs, it often indicates that the tumor cells have successfully evaded several “host‐dependent” challenges, such as remodeling the native stromal environment of primary and metastatic organs, inducing vascular and neural infiltration, achieving immune escape at the primary site, and modifying the immune landscape at the metastatic site [5]. In clinical practice, the latency period between the diagnosis of the primary tumor and the detection of liver metastasis varies greatly among tumor types. Even among liver metastatic tumors, the routes of metastasis, growth patterns (GPs) in the liver, and the resulting liver damage differ [2, 6, 7]. However, from an organ perspective, metastatic tumors of different origins undergo highly similar biological processes in the liver and induce many of the same hepatic responses [8]. Therefore, recognizing the heterogeneity of metastatic tumor cells from different origins while exploring the common features of liver metastases should be a fundamental consensus in the study of liver metastasis therapy.

The “seed and soil” theory provides a fundamental hypothetical model for understanding the process by which metastatic tumors colonize target organs, highlighting the role of positive interactions between malignant cells and the tumor microenvironment (TME) in the establishment and growth of metastases [9]. Additionally, the “mechanical metastasis” theory and some classic research suggest that local dissemination is more influenced by physical anatomy, while distant metastasis colonization is often attributed to the organ specificity of malignant cells [10, 11, 12]. The prevailing view is that metastasis is a complex, dynamic, and continuous biological process. It involves the accumulation of invasive phenotypes and the reshaping of the TME at primary lesions. It also includes the adaptive survival of tumor cells, their dynamic transendothelial behavior during dissemination, and their invasive growth at ectopic metastatic sites.

As an organ responsible for vital physiological functions, the emergence and progression of liver tumors significantly compromise liver function, worsening the patient's condition [13, 14]. Liver metastasis is generally associated with a decline in the 5‐year survival rate and a deterioration in quality of life [15]. Patients with liver metastases also demonstrate a higher degree of treatment resistance compared with those without liver metastases [16, 17]. Even after successful surgical resection, recurrence often occurs within a relatively short period [18, 19]. With few exceptions, current treatment strategies continue to struggle in controlling the progression of liver metastases.

In this comprehensive review, we explore why the liver is a preferred site for metastasis in many tumors. We discuss the changes in metastatic tumor cells during metastasis and, from an organ‐specific perspective, propose three liver reactive stages and their corresponding characteristics. We also detail the functions and spatial redistribution of both inherent and recruited cells associated with liver metastasis, emphasizing their spatiotemporal plasticity. Furthermore, we discuss the main strategies and challenges in the current management of liver metastases.

## 2 Metastatic‐Friendly Features of Liver

### 2.1 Anatomic and Metabolic Characteristics

An anatomical consensus is that the liver, as the first organ to filter blood from the portal vein and hepatic artery, has an abundant blood supply [20]. The portal vein is a common pathway for metastasis of gastrointestinal tumors, while the hepatic artery is commonly utilized by lung and breast tumor metastases. The liver's slow peripheral blood flow provides ample opportunity for malignant cell colonization. Some tumor cells can also migrate retrogradely to the liver through the lymphatic network, while the numerous nerve fibers accompanying the arteries and bile ducts may serve as natural conduits for tumor metastasis [21, 22, 23, 24]. Additionally, the liver's considerable volume within the abdominal cavity allows close contact with adjacent organs, such as the stomach, gallbladder, pancreas, and kidneys, facilitating direct invasion by malignant tumors.

The liver, as the primary organ governing the body's energy balance, provides an abundant energy reserve for metastatic tumor cell growth [25]. The liver lobule, the basic functional unit of the liver, features a highly structured arrangement, a fixed blood flow direction, and sequential enzymes of the metabolic cascade along the portal–mesenteric axis, resulting in varying concentrations of oxygen and metabolites within localized areas [26]. Oxygen concentration is higher around the portal vein, where hepatocytes predominantly perform processes such as gluconeogenesis, fatty acid oxidation, and protein synthesis and secretion. By contrast, the area around the central vein has lower oxygen levels, with lipogenesis and glycolysis being relatively active [27, 28, 29]. Tumor cells, highly reliant on metabolic reprogramming to optimize energy uptake, also influence stromal and immune cell functions by locally releasing metabolic products [30, 31]. Metastatic tumor cells undergo organ‐specific metabolic adaptations, enabling them to overcome the unique barriers to liver colonization and establish a metastatic tissue tropism phenotype [32, 33]. Specifically, metastatic tumor cells exhibit higher activity levels of glycolysis, lipid metabolism, and nucleotide metabolism compared with primary tumor cells [34, 35, 36, 37]. However, whether metabolic preferences influence metastatic cell colonization sites and the mechanisms by which tumor cells remodel liver metabolic TME remain unclear. Notably, despite these metabolic adaptations, liver metastatic tumors retain a metabolic profile more similar to their primary tumor of origin than to primary liver tumors [38].

Extrahepatic tumors induce liver infiltration of myeloid cells, which, through IL6–pSTAT3 immune–hepatocyte crosstalk, leads to systemic and tissue‐specific metabolic changes that promote self‐liver metastasis [39]. Additionally, liver sinusoidal endothelial cells (LSECs), which typically regulate transendothelial transport of substances and cells, may experience compromised fenestration and constriction functions under certain pathological conditions, such as the accumulation of specific macronutrients or toxins, further increasing the likelihood of metastatic colonization [40]. In pathological states such as nonalcoholic fatty liver disease (NAFLD), the liver's metabolic homeostasis is disrupted [41, 42]. Altered pre‐existing metabolic compartmentalization and local accumulation of specific metabolic enzymes and products may enhance the liver's adaptation to tumor metastasis [26].

### 2.2 Default Immunosuppressive Inclination

The liver, the largest collection of phagocytic cells, acts as a vital immunological barrier between the human body and the external environment. The liver is constantly exposed to various mediators that threaten immune balance. It receives inputs from the portal vein, including gut‐derived pathogens, microbe‐derived molecules, pathogen‐associated molecular patterns (e.g., toll‐like receptor 3 [TLR3] and TLR9 ligands), toxins, nutrients, and metabolites, as well as from the hepatic artery, such as systemic infections and antibodies. The liver typically maintains a restrained immune response but can generate a rapid and robust immune response under appropriate conditions [43]. Human liver immune cells comprise diverse subpopulations with distinct functions, and certain subpopulations of Kupffer cells (KCs) share similar gene expression and pathways with endothelial cells [27, 44]. This finding suggests that the liver's immune profile is maintained through numerous dynamic interactions among various cell populations.

As the predominant subtype of tissue‐resident macrophages in the liver, KCs play a crucial role in maintaining immunosuppressive orientation. KCs express various scavenger receptors, complement receptors, and TLRs and present antigens via major histocompatibility complex (MHC) I, MHC II, and CD1d [45, 46]. Upon activation, KCs release a range of acute‐phase proteins, cytokines, and chemokines that activate intrahepatic resident natural killer (NK) cells and natural killer T (NKT) cells. In some cases, KCs recruit additional immune cells to infiltrate the liver [45, 47]. Additionally, KCs suppress the antigen‐presenting function of dendritic cells (DCs) and LSECs by releasing molecules such as prostaglandins and IL‐10; KCs also induce functional tolerogenic T‐cell responses [43, 46, 48]. KCs engulf activated or apoptotic neutrophils, thereby limiting their potential to induce inflammation [49].

LSECs constitute the direct physical barrier for substance exchange between the liver parenchyma and the blood, outnumbering liver‐resident immune cells by more than twofold [50]. In addition to actively regulating molecular exchanges, LSECs express various pattern recognition receptors, MHC I and MHC II, costimulatory molecules (CD80, CD86), and adhesion molecules (ICAM), enabling them to present antigens directly to T cells [51, 52]. LSECs inhibit the antigen‐presenting function of other parenchymal cells and dynamically regulate T‐cell immune tolerance [53, 54, 55]. Specifically, LSECs can maintain CD8+ T cells in a state of continuous proliferation without effector function (cytokine production) by increasing the expression of coinhibitory B7‐H1 while not expressing costimulatory CD80/86 molecules [56, 57]. Additionally, some pathogen‐derived molecules or immune mediators reduce the expression of MHC and costimulatory molecules in LSECs, further limiting their ability to initiate immune responses [58, 59]. These LSEC‐mediated regulatory mechanisms are essential for maintaining normal liver immune homeostasis.

Hepatocytes have been found to participate in the innate immune response, reducing viral replication and inhibiting the secretion of inflammatory factors [60, 61]. Although not the primary antigen‐presenting cells, hepatocytes can present antigens to the adaptive immune system by expressing various immune receptors. Additionally, hepatocytes contribute to T‐cell activation through direct physical contact and regulate adaptive immune function by engulfing CD4+ T cells or converting CD4+ T cells into Treg cells [62, 63, 64, 65].

NK cells play a crucial role in immune surveillance; but those in liver tend to be typically hyporesponsive and perform limited functions [66]. Inflammatory monocytes infiltrate the liver and differentiate into myeloid‐derived suppressor cells (MDSCs) under the influence of TGF‐β [67]. MDSCs bind to NCR3 receptors to inhibit NK‐cell function [68]. They also highly express CD84 and produce reactive oxygen species and arginase 1 (Arg1), further suppressing T‐cell function [69, 70]. Additionally, Treg cells produce various anti‐inflammatory cytokines and engage in the PD‐1 inhibitory pathway to effectively suppress T‐cell‐mediated immune responses [71, 72].

## 3 Panorama of Liver Metastasis

### 3.1 Active Plasticity of Metastatic Tumor Cells

The widely accepted view is that tumor progression is not merely an increase in volume but a continuous process of mutation and selection, during which tumor cells adapt to survival pressures such as hypoxia or nutrient competition. This process results in a more genetically and phenotypically heterogeneous population within the tumor [1]. This concept has also been extended to explain tumor metastasis, a systemic disease driven by high aggressive tumor cells [73]. Numerous signaling pathways are aberrantly activated within tumor cells, as summarized in Table 1, equipping them to depart from the primary lesion.

**TABLE 1:** Classic signaling pathways involved metastasis and their corresponding mechanisms of primary tumor cells.

Signaling pathways	Functions	Mechanisms	References
TGF‐β	Enhances invasive capacity	Drives EMT process	[74]
		Induces activation of myCAFs	[75]
	Modulates immunity	Drives immune evasion	[76, 77]
		Drives NK cell metabolic dysfunction	[78]
		Induces NETs	[79]
	Stromal remodeling	Promotes angiogenesis	[77, 80]
		Promotes lymphangiogenesis	[81]
	Metabolic reprogramming	Regulates cholesterol metabolism	[82]
Wnt/β‐catenin	Enhances invasive capacity	Drives EMT process	[83, 84]
		Activates planar cell polarity	[85]
		Maintains stemness	[86]
	Modulates immunity	Drives immune evasion	[87]
		Induces systemic inflammation	[88]
	Stromal remodeling	Regulates extracellular matrix degradation	[89]
		Promotes angiogenesis	[90]
		Promotes lymphangiogenesis	[91]
	Metabolic reprogramming	Upregulates glycolytic enzyme ALDOC expression	[92]
MAPK	Enhances invasive capacity	Drives EMT process	[93, 94]
		Maintain stemness	[95]
	Modulates immunity	Drives immune evasion	[96]
	Stromal remodeling	Promotes angiogenesis	[97]
		Promotes lymphangiogenesis	[98]
		Promotes perineural invasion	[99]
	Metabolic reprogramming	Promotes glycolysis	[99]
PI3K/AKT	Enhances invasive capacity	Drives EMT process	[100]
		Maintains stemness	[101]
		Promotes CTCs formation	[102]
	Modulates immunity	Drives immune evasion	[103]
		Promotes neutrophil recruitment and reprogramming	[104]
		Promotes resistance against NK cell killing	[105]
	Stromal remodeling	Promotes angiogenesis	[106, 107]
		Promotes lymphangiogenesis	[108]
		Promotes perineural invasion	[109]
	Metabolic reprogramming	Regulates nutrient transporters, metabolic enzymes and key components of metabolic pathway	[110]
JAK/STAT	Enhances invasive capacity	Drives EMT process	[111, 112]
		Maintains stemness	[113]
	Modulates immunity	Drives immune evasion	[114, 115]
	Stromal remodeling	Promotes angiogenesis	[116]
		Promotes perineural invasion	[117]
NF‐κB	Enhances invasive capacity	Drives EMT process	[118]
		Maintains stemness	[119]
	Modulates immunity	Drives immune evasion	[120]
		Promotes macrophage‐mediated immune suppression	[121]
	Stromal remodeling	Promotes angiogenesis	[122]
		Promotes lymphangiogenesis	[123]
	Metabolic reprogramming	Regulates glucose metabolism	[124, 125]

Abbreviations: EMT, epithelial‐to‐mesenchymal transition; myCAFs, myofibroblastic cancer‐associated fibroblasts; NK cell, natural killer cell; NETs, neutrophil extracellular traps; ALDOC, aldolase C; CTCs, circulating tumor cells.

Compared with primary tumor cells, metastatic tumor cells often have higher levels of chromosomal instability and a higher proportion of clonal mutations, as well as high frequencies of whole‐genome duplication and TP53 mutations [126, 127]. Some specific genes or pathways are also detected to be abnormally active mainly in metastatic tumor cells [128, 129]. The PTEN loss, RB1 loss, and APC mutations may explain the liver tropism of tumor metastasis [126]. However, some studies have found that primary tumors and liver metastases share a high degree of similarity in somatic mutations, copy number variations, and transcriptome profiles [130, 131, 132]. In some tumors, the frequency of KRAS and TP53 mutations in the metastatic tumor cells is the same as in the primary tumor cells [133]. These findings are more consistent with the parallel progression model, which suggests that the dissemination of tumor cells may occur in the early stage of the tumor, or even in the precancerous stage; different tumor clones may concurrently colonize multiple secondary sites.

In addition to the “seed,” changes in the “soil” may also be of significant importance in the process of metastasis. Recently, Mei et al. [134] have found that common germline variants may underlie tumor metastatic outcomes. The common missense variant rs562556 in the proprotein convertase subtilisin/kexin type 9 (PCSK9) gene promotes breast cancer metastasis and is associated with reduced patient survival. Mechanistically, this PCSK9 mutant degrades tumoral low‐density lipoprotein receptor‐related protein 1 receptors, consequently leading to the induction of prometastatic genes [134]. While further evidence is required to substantiate the hypothesis, this discovery indicates that individual genetic backgrounds should be thoroughly taken into account in the study of tumor metastasis.

Tumor cells must continuously adapt to the TME of primary lesions, local migratory niches, and metastatic lesions to survive [135]. Active plasticity is evident in the exacerbation of heterogeneity within genetically diverse metastatic tumor cell populations, promoting metastasis progression. Research has shown that Lgr5+ stem cells play a vital role in the progression of primary colon cancer [136]. However, circulating tumor cells (CTCs) are predominantly Lgr5−. After liver colonization, influenced by environmental factors such as hepatocyte growth factor (HGF) and fibroblast growth factor (FGF), metastatic tumor cells re‐express Lgr5 to support their growth in the liver [137]. L1CAM is not required for tumor initiation but is necessary for primary tumor propagation and liver metastatic colonization [138]. When epithelial integrity is lost, malignant progenitor cells adopt a highly plastic regenerative phenotype characterized by migration, anoikis evasion, and L1CAM‐dependent ectopic regrowth [138, 139]. Glutathione S‐Transferase Theta 1 (Gstt1) is uniquely required for dissemination and ectopic growth of metastatic tumor cells, but it is dispensable for primary tumor. Gstt1 is expressed in latent metastatic tumor cells and is also retained within a subpopulation of slow‐cycling metastatic tumor cells. Mechanistically, Gstt1 interacts with intracellular fibronectin and modifies it via glutathione conjugation, thereby influencing the deposition of fibronectin into the local niche through a mechanism that is crucial for driving metastasis [140]. Additionally, during metastasis, tumor cells lose intestinal cell identities and reprogram into a highly conserved fetal progenitor state, acquiring the capacity for noncanonical differentiation into divergent squamous and neuroendocrine‐like states. Metastatic tumor cells, unlike their primary counterparts, exhibit an enhanced ability for cell‐autonomous multilineage differentiation. This allows them to diversify into a range of intestinal and nonintestinal lineages as a strategy to evade the cytotoxic effects of cancer therapies [141]. The plasticity mechanisms in metastatic tumor cells also involve epigenetic and posttranslational modifications. The intricate interplay between signaling pathways and heritable modifications plays a significant role throughout metastasis [142, 143, 144]. Some epigenetic therapies have shown promising effects in treating metastasis, although they face challenges in achieving therapeutic specificity and precise delivery [145].

In summary, understanding metastasis from the perspective of the active plasticity of metastatic tumor cells and exploring the mechanisms by which metastatic heterogeneity is generated over smaller temporal dimensions may be key to developing future antimetastatic treatments.

### 3.2 Three Response Phases of Liver to Metastasis

**FIGURE 1:**
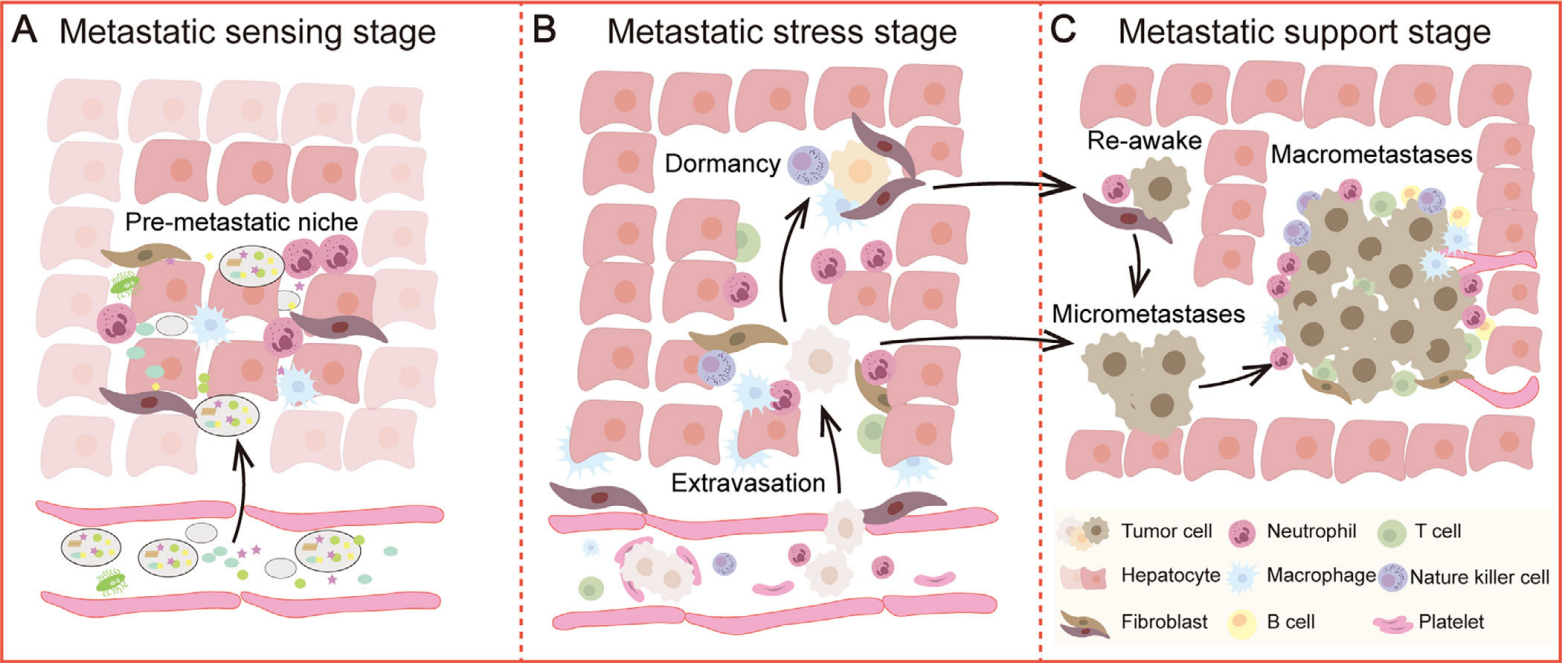
Three phases of liver metastasis based on the liver's response. (A) Metastatic sensing stage: The liver senses factors derived from both tumor and nontumor sources, accompanied by a massive infiltration of immune cells. The premetastatic niche is formed, preparing for the arrival of metastatic tumor cells. (B) Metastatic stress stage: The liver parenchyma comes into contact with metastatic tumor cells that have crossed the vascular endothelium. Utilizing immune and nutrient‐deprived mechanisms, the liver eliminates the majority of these metastatic tumor cells; only a small fraction manage to enter a state of dormancy. (C) Metastatic support stage: Reactivated metastatic tumor cells induce functional differentiation and spatial redistribution of both resident and recruited cells within the liver, thereby fostering their own growth. Consequently, the liver forfeits its capacity to combat metastasis, supporting the progression of metastasis at the expense of its own functions.

Many studies on tumor metastasis have focused on the metastatic potential of primary tumor cells and the primary TME. However, new therapies are almost first tested in metastatic patients [146]. Due to the many limitations of extrapolating conclusions from primary tumors [135, 141], researchers have shifted their attention to the intrinsic and acquired characteristics of metastatic tumors in search of opportunities for predicting and treating metastasis. The liver is not passively invaded by metastatic tumor cells, but rather makes a series of adaptive responses under the individual genetic backgrounds, comorbidities, and tumor‐related pathophysiological disorders. Consequently, liver metastasis can be divided into three phases based on the liver's response: the metastatic sensing stage, the metastatic stress stage, and the metastatic support stage (Figure 1).

#### 3.2.1 Metastatic Sensing Stage

Because of its constant exposure to large volumes of systemic blood, the liver acts as an early biosensor for both endogenous and exogenous molecules, regardless of disease type. It senses tumor‐derived molecules, including cytokines, soluble proteins, and extracellular vesicles, as well as factors from non‐neoplastic components such as the gut microbiome and alcohol, as outlined in Table 2. Non‐neoplastic factors can influence the reactivity of liver parenchymal cells to tumor‐derived molecules. Obesity, surgery, liver diseases, pregnancy, diet, and medication also affect the liver's susceptibility to metastasis, although these factors do not necessarily result in metastasis [42, 147, 148]. Communication between organs is mediated by hormones, secreted factors, and nerves, such as in the brain–liver axis [149], lung–liver axis [150], spleen–liver axis [151], and gut–liver axis [152]. This crosstalk helps maintain organ homeostasis and suppress disease progression. Similarly, an organ‐level regulatory mechanism is also thought to exist in liver metastasis, influencing the malignant process by altering certain properties of the liver [147, 153]. Experimental animal models have shown specific pathological changes in the liver that occur before metastatic tumor cells arrive. Several molecular markers, including LOX, serum amyloid A1/2 (SAA1/2), TIMP‐1, MIF, SDF‐1, fibronectin, TGF‐β1, uPA, and S100A8, as well as the macrophage markers CD68, CD11b, and CD163 and the neutrophil marker Ly‐6G, have been used as histological indicators to identify sites of metastatic malignant cell colonization [154, 155]. Although similar data from clinical patients are unavailable, these findings suggest a dramatic response of the liver in early sensing metastasis.

**TABLE 2:** Metastasis‐associated factors sensed by the liver.

Types	Molecules	Mechanisms	References
Cytokines	IL‐6	Activates STAT3 signaling and releases SAA1/2 by hepatocytes	[156]
	TGF‐β	Induces transformation and activates chemokine signaling in HSCs to recruit MDSCs	[157]
	CCL2	Activates Wnt/β‐catenin signaling in HSCs	[158]
	CXCL1	Recruits CXCR2+ MDSCs	[159]
Soluble protein	TIMP‐1	Recruits neutrophil by SDF‐1/CXCR4 axis	[155]
	MIF	Activates KCs and releases TGF‐β	[160]
	LBP	Activates TLR4/NF‐κB signaling in KCs and releases TGF‐β	[161]
	GRP78	Inhibits DCs activation, induces M2‐like polarization, and elevates TGF‐β level	[162]
Extracellular vesicles	miRNA miR‐25‐3p	Promotes VEGFR2, ZO‐1, occludin, and Claudin5 in endothelial cells and induces vascular leakiness	[163]
	miRNA‐181a‐5p	Targets SOCS3 and activates IL‐6/STAT3 signaling in HSCs, promotes the CCL20/CCR6/ERK1/2/Elk‐1/miRNA‐181a‐5p positive feedback loop; inhibits SP3 in KCs and releases TGF‐β	[164, 165]
	miRNA‐21‐5p	Induces macrophage polarization toward an IL‐6‐secreting proinflammatory phenotype by activating TLR7	[166]
	miRNA‐135a‐5p	Facilitate the metastatic tumor cell adhesion and suppress CD4+ T cells	[167]
	miRNA‐934	Promotes M2 macrophage polarization and activates the CXCL13/CXCR5/NFκB/p65/miRNA‐934 positive feedback loop	[168]
	miRNA‐519a‐3p	Induces M2 macrophage polarization and induces angiogenesis	[169]
	miRNA‐9‐5p	Links oxysterol metabolism and KCs polarization	[170]
	tRF‐GluCTC‐0005	Activates WDR1 in HSCs and recruits MDSCs	[171]
	Integrin αvβ5	Activates src in KCs and promotes cell migration genes and *S100* genes expression	[172]
	ITGBL1	Activate TNFAIP3‐NF‐κB in HSCs.	[173]
	circ‐0034880	Activates SPP1^high^CD206^+^ protumor macrophages	[174]
	HSPC111	Regulates lipid metabolism of HSCs and promotes EMT by CXCL5–CXCR2 axis	[175]
	CD36	Regulates immune cell invasion and extravasation of microvesicles	[176]
	CD44v6/C1QBP	Activates HSCs and promotes liver fibrosis	[177]
	Netrin‐1	Activates HSCs and promotes retinoic acid secretion	[178]
	ANGPTL1	Inhibits JAK2–STAT3 signaling in KCs	[179]
	Fatty acid	Activates KCs and promotes TNF secretion	[13]
	DNA	Activates DNA damage responses in KCs, rewires cytokine production, and promotes the formation of TLS	[180]
Other factors	NETs	Promotes the adhesion of CTCs and activates HSCs	[181, 182]
	Alcohol	Disrupts the homeostasis of hepatocytes and mediates neutrophil‐mediated T cell exhaustion	[183]
	Enterotoxigenic *B. fragilis*	Induces systemic inflammation, regulates the immune, and promotes EMT	[184]
	Fusobacterium nucleatum	Induces systemic inflammation and regulates the immune	[185]
	Escherichia coli	Favors the recruitment of metastatic cells	[186]
	Capsaicin	Promotes bacteria translocation to liver and regulates the recruitment of NKT cells	[148]

Abbreviations: STAT3, signal transducer and activator of transcription 3; SAA1/2, serum amyloid A 1/2; HSCs, hepatic stellate cells; MDSCs, myeloid‐derived suppressor cells; TIMP‐1, tissue inhibitor of metalloproteinases‐1; SDF‐1, stromal cell‐derived factor‐1; MIF, migration inhibitory factor; KCs, Kupffer cells; LBP, lipopolysaccharide binding protein; TLR4, Toll‐like receptor 4; GRP78, glucose‐regulated protein 78; DCs, dendritic cells; VEGFR2, vascular endothelial growth factor receptor 2; ZO‐1, zonula occludens‐1; SOCS3, suppressors of cytokine signaling 3; SP3, specificity protein 3; WDR1, WD repeat domain 1; SPP1, phosphoprotein 1; EMT, epithelial‐to‐mesenchymal transition; ANGPTL1, angiopoietin‐like protein 1; TNF, tumor necrosis factor; TLSs, tertiary lymphoid structure; CTCs, circulating tumor cells; NKT cells, natural killer T cells.

CTCs are direct vehicles of metastasis, exhibiting significant heterogeneity in molecular phenotype, transcriptome, and cytological characteristics. CTCs that have detached from the primary tumor must overcome various environmental pressures in circulation, including immune killing, anoikis, oxidative stress, blood fluid shear forces, and oxygen/nutrient deprivation. Ultimately, only a small fraction of CTCs successfully reach the liver and attach to the walls of small blood vessels to gain access for invasion [145, 187, 188]. The presence of CTCs can be detected in circulation at very early stages of tumorigenesis [189]. While the half‐life of CTCs is consistent across different tumor types, the rate of CTC production varies. CTCs can trigger metastasis in healthy mice even in the absence of primary tumors; however, the emergence of CTCs does not guarantee metastasis [190]. In addition to molecular mediators, specific characteristics of CTCs influence their propensity for liver seeding. For instance, hybrid CTCs expressing both epithelial and mesenchymal markers predominantly colonize the liver, whereas mesenchymal CTCs are more likely to metastasize to extrahepatic sites [189]. Furthermore, elevated expression of CD133 and CD110 on cell surfaces [191, 192], along with distinct metabolic preferences for lysine and folate [193], have been identified as significant factors contributing to the high liver metastatic potential of CTCs.

#### 3.2.2 Metastatic Stress Stage

High expression of aspartate β‐hydroxylase in tumor cells activates the Notch cascade and induces the formation of exosomes containing matrix metalloproteinases (MMPs), which promote the transendothelial migration [194]. Amphoterin‐induced gene and open reading frame 2, derived from tumor cells, is internalized by LSECs, significantly enhancing their capacity to adhere to CTCs [195]. In vitro experiments have shown that aberrant activation of the c‐Met pathway facilitates the extravasation of metastatic tumor cells, while blocking c‐Met phosphorylation effectively inhibits this process [196]. Prostaglandin E synthase enzyme 3 (PTGES3) acts as an invasion suppressor by inhibiting phosphofructokinase to regulate glycolysis. Low expression of PTGES3 in metastatic tumor cells leads to excessive activation of glucose oxidation, ultimately facilitating tumor cell entry into the hepatic parenchyma [197]. Active necroptosis in metastatic tumor cells recruits macrophages and induces the formation of macrophage extracellular traps (METs), accelerating tumor adhesion to endothelial cells via the CXCL8–ICAM‐1 axis [198]. Metastatic tumor cells also exhibit high expression of heterogeneous nuclear ribonucleoprotein AB (hnRNPAB), which stabilizes MYC mRNA and promotes neutrophil recruitment at extravasation sites through the secretion of CXCL8 [199]. Neutrophils facilitate extravasation via mechanisms such as degranulation and the release of neutrophil extracellular traps (NETs) [200, 201]. Additionally, exocrine factors, including autocrine and matrix‐derived CXCL12, serine protease inhibitor B5, and cystatin B, have been found to enhance the transendothelial migration of metastatic tumor cells [202, 203].

In addition, invadopodia are specialized membrane protrusions formed by metastatic tumor cells that degrade the basement membrane, facilitating the transmembrane process. Studies have shown that soluble platelet‐derived growth factor subunit B released by metastatic tumor cells activates hepatic stellate cells (HSCs) within the TME of liver metastases. Activated HSCs, in turn, promote the formation of invadopodia through the HGF/MET/PI3K/AKT signaling pathway [204]. The chemokine CCL7 not only recruits macrophages but also regulates invadopodia maturation via the CCR3 signaling pathway [205].

The heterotypic adhesion between tumor cells and endothelial cells is facilitated by a distinct sequence of signaling events involving mediators such as selectins, cadherins, and integrins [206, 207, 208]. Long noncoding RNAs are significant regulators of mammalian transcription. lncGALM, which is highly expressed in metastatic tumor cells, acts as a molecular sponge for miRNA‐200 to promote the expression of epithelial‐to‐mesenchymal transition (EMT)‐related transcription factors ZEB1 and ZEB2. lncGALM also contributes to LSEC apoptosis by stabilizing IL‐1β mRNA in metastatic tumor cells, thereby inducing endothelial barrier opening to facilitate transendothelial tumor cell migration [209]. Metastatic tumor cells also secrete IL‐23, which induces TNF‐α production in LSECs, upregulating MMP9 and ICAM1 expression and promoting the formation of endothelial cell gaps [210]. The integrity of the vascular endothelium is further regulated by immune cells. For example, IL‐22 derived from NKT cells acts on LSECs, enhancing endothelial permeability and tumor cell transmigration by inducing endothelial aminopeptidase N [211].

Platelets participate in the invasion and metastasis of tumors through various mechanisms. Due to changes in their counts, molecular expression, and transcriptional profile, they have also been found to be good biomarkers for monitoring metastasis [212]. The role of platelets in metastatic tumor cell extravasation remains controversial. Platelets form aggregates with tumor cells and are activated by tumor‐derived podoplanin. Once activated, platelets release TGF‐β, promoting EMT and extravasation of metastatic tumor cells [213]. Activated platelets also release ATP, which binds to P2Y_2_ receptors on LSECs, modulating their cytoskeleton and endothelial junctions [214]. However, some studies suggest that thrombin‐activated platelets protect LSECs from tumor‐derived damage by forming tight junctions mediated by VCAM‐1, thereby inhibiting metastatic tumor cell extravasation [215].

The liver eliminates most successfully extravasated metastatic tumor cells through innate immunity or deprivation of oxygen and nutrients [26]. In response, metastatic tumor cells often enter a state of dormancy within ectopic niches and can be reawakened under specific conditions. Dormant tumor cells continue to accumulate mutations and epigenetic alterations, resulting in more challenging disease control. While metastatic tumor cells escape anoikis and immune killing, this occurs at the expense of self‐renewal. Dormancy programs are regulated not only by tumor‐intrinsic signaling but also by the local niche. A classical mechanism for dormancy regulation involves a balance between intracellular p38α/β and ERK1/2, which is controlled by urokinase fibrinogen activator receptor, TGF‐β, integrins, and other soluble molecules [216]. Additionally, factors such as interactions between NK cells and HSCs, cytokines released by T lymphocytes, the biomechanical properties of the stroma, systemic inflammation, and the metabolic environment influence the survival of dormant tumor cells in the liver [217, 218, 219, 220, 221].

#### 3.2.3 Metastatic Support Stage

Under certain conditions, a subset of dormant tumor cells can re‐enter the cell cycle, inducing stromal remodeling, angiogenesis, and immunosuppression within the niche, ultimately leading to the formation of detectable metastatic lesions. While the duration of dormancy in metastatic tumor cells cannot be definitively determined, studies suggest that individual genetic background, surgery, medication, and metabolic disorders are significant factors in shortening the latency period [222, 223, 224]. The critical mutations or epigenetic modifications that reawaken dormant tumor cells in the liver remain poorly understood. However, factors such as hypoxia, local inflammation, immune stimulation, mechanical stress, and microorganisms may directly drive this activation process.

Research has shown that activation of the stimulator of interferon genes (STING) pathway promotes dormant tumor cells to re‐enter the cell cycle. During subsequent progression, the STING promoter and enhancer become hypermethylated, affecting metastasis by regulating NK‐cell and T‐cell functions [225]. The transcription factor NRF2 plays a critical role in reactivated dormant tumor cells. NRF2 signaling induces metabolic reprogramming at the transcriptional level, re‐establishing redox homeostasis and enhancing de novo nucleotide synthesis [226]. NETs cleave laminin in the niche through neutrophil elastase and MMP9, and the subsequent reorganization of laminin activates the proliferation of dormant tumor cells via integrin α3β1 signaling [182]. Another matrix protein, MMP2, also reinitiates dormant tumor cell proliferation through fibrinolysis [227]. Endothelial cells contribute to the transition between tumor cell dormancy and activation. In vitro experiments have shown that the dormant state is disrupted by neovascular sprouting. Mechanistically, endothelial tip cells promote the reactivation of dormant tumor cells by releasing active TGF‐β1 and periostin [228].

The invasive growth of metastatic tumor cells involves a variety of complex biological processes, including immune evasion, metabolic reprogramming, and remodeling of the liver metastatic niche (LMN), ultimately causing severe liver function damage and forming life‐threatening macrometastases. Liver metastases grow according to four different histological patterns, termed the “desmoplastic,” “pushing,” “replacement,” and “mixed” GPs. Briefly, in the desmoplastic GPs, metastases are separated from the liver parenchyma by a desmoplastic stroma. The mesenchyme is densely infiltrated with lymphocytes, and there is no direct contact between the metastatic tumor cells and the surrounding liver cells. In the pushing GPs, metastases directly compress the surrounding liver tissue. There is no desmoplastic stroma, and only a mild inflammatory infiltrate is present within the border of metastases. In the replacement GPs, metastatic tumor cells replace the space of hepatocytes, preserving the original structure of the liver parenchyma. There is close cell–cell contact between tumor cells and hepatocytes, with no desmoplastic stroma or inflammatory infiltrate. The mixed GP is a combination of the aforementioned GPs. Studies have found that metastases with a pushing GP are characterized by angiogenesis, which is associated with aggressive biology and a poor clinical outlook [229]. Chemotherapy and metastatic tumor cell ablation induce the formation of desmoplastic stroma, potentially halting metastasis progression and improving patient survival [230]. The mechanisms underlying the formation of differential GPs of metastases remain elusive; factors such as mechanical memory, tumor–hepatocyte interactions, metastatic routes, and hypoxia only partially explain this phenomenon [229, 230, 231, 232].

## 4 Cells Involved in Liver Metastasis

**FIGURE 2:**
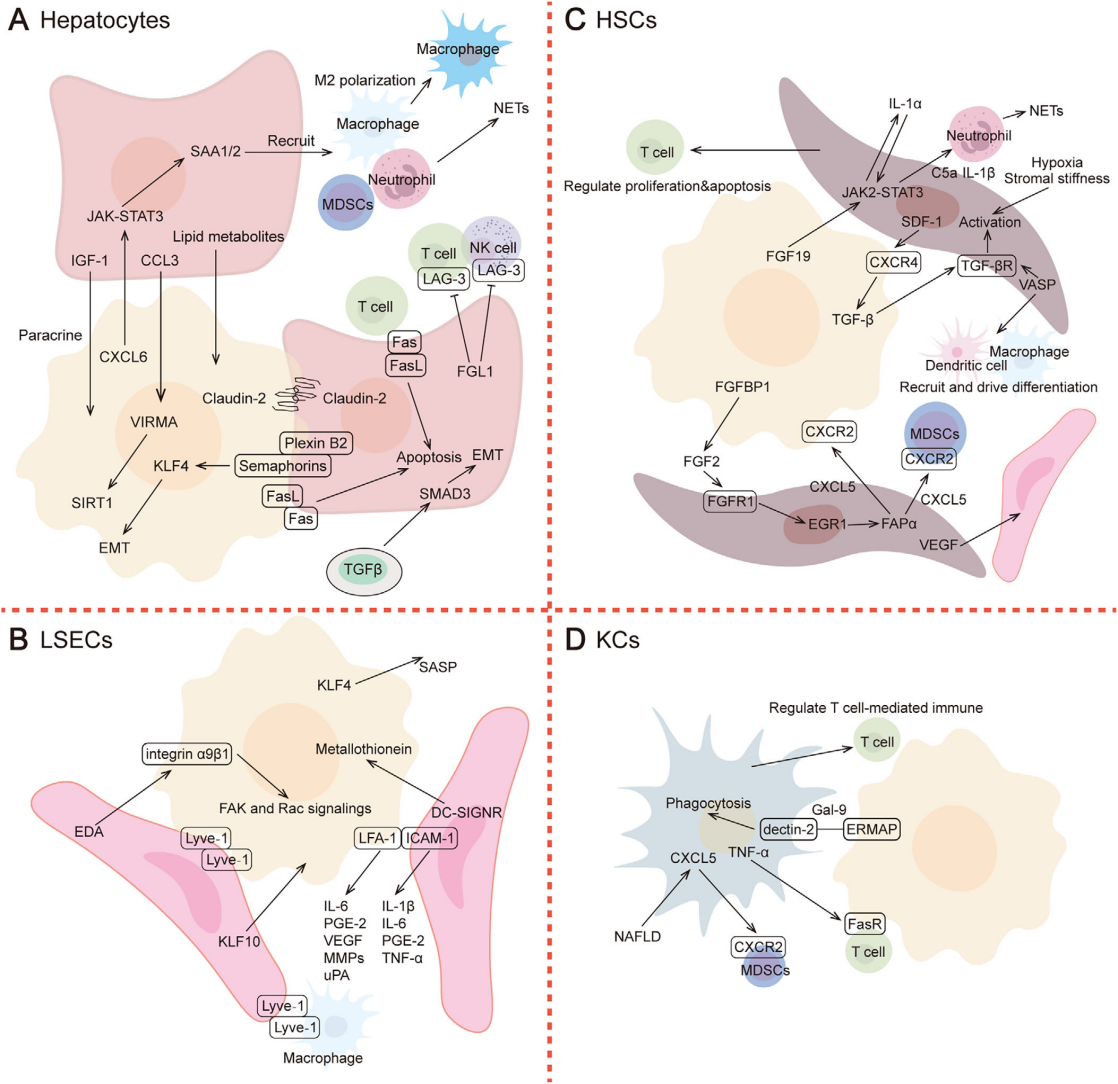
Functional characteristic and molecular mechanisms of resident cells involved in liver metastasis. (A) Hepatocytes play multiple complex roles, including promoting metastatic tumor cell colonization and proliferation, as well as regulating immune responses and providing nutritional support. (B) LSECs not only participate in the extravasation of metastatic tumor cells but also continuously promote metastatic progression. (C) HSCs promote tumor invasion, angiogenesis, immune regulation, and treatment resistance. (D) KCs exhibit dynamic plasticity, inhibiting metastasis in the early stages and promoting it in the late stages.

**FIGURE 3:**
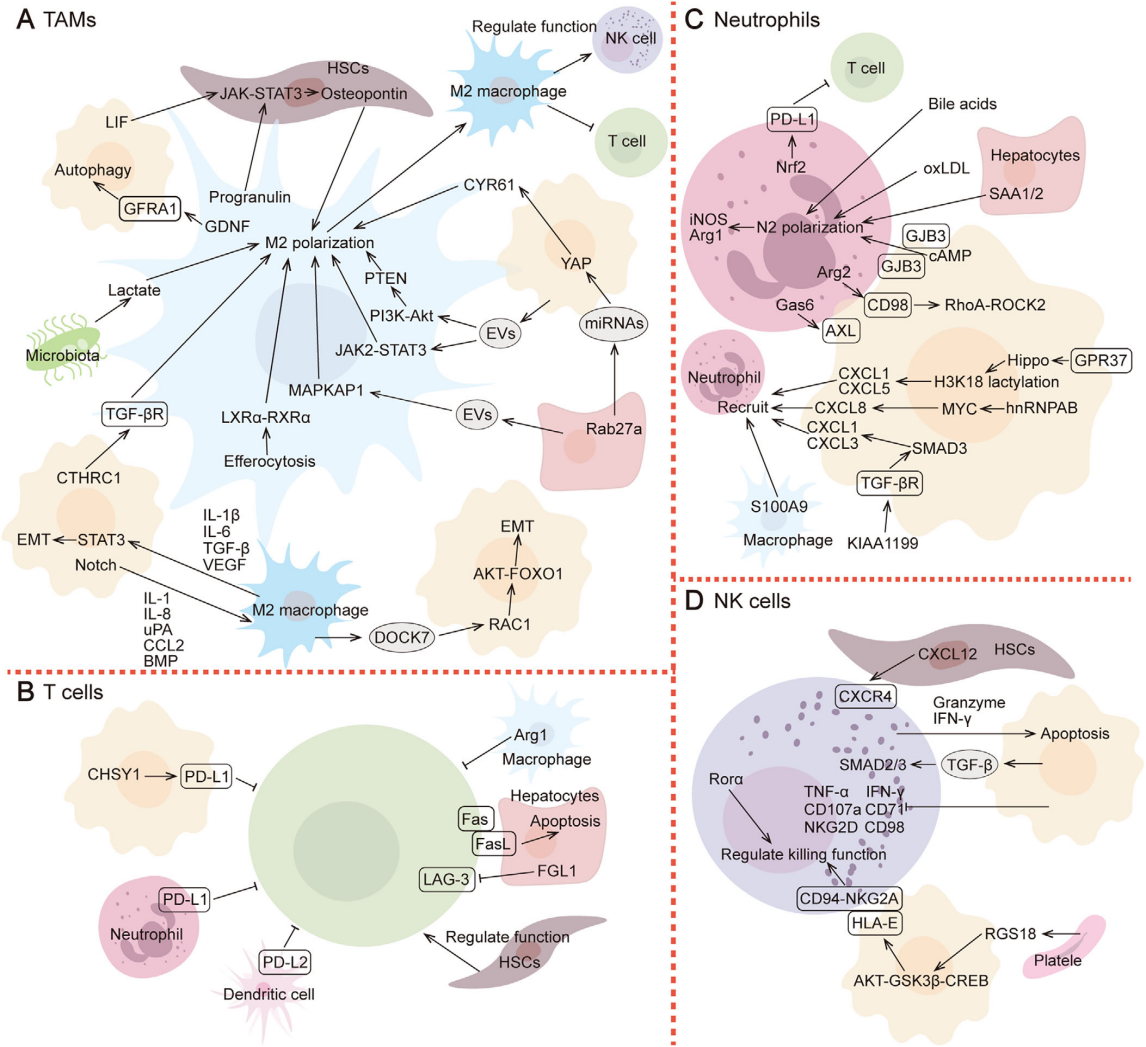
Functional characteristic and molecular mechanisms of recruited cells involved in liver metastasis. Liver metastasis is often accompanied by the infiltration of a large number of recruited cells, including TAMs (A), T cells (B), neutrophils (C), and NK cells (D). These immune cells exhibit rich heterogeneity and dynamic functional plasticity. They always lose their cytotoxic functions and instead play a role in promoting metastasis.

Liver metastasis involves interactions between various resident cells (e.g., hepatocytes, LSECs, HSCs, and KCs) and recruited cells [e.g., tumor‐associated macrophages (TAMs), neutrophils, NK cells, and T cells] (Figures 2 and 3). Delving deeper into the functional and spatial characteristics of cells involved in liver metastasis helps identify effective therapeutic targets.

### 4.1 Functional Characteristics

#### 4.1.1 Hepatocytes

The contact between hepatocytes and metastatic tumor cells is an early event in metastasis. In vitro experiments have demonstrated that tumor cells can form physical protrusions to adhere to hepatocytes, a process potentially facilitated by integrins and claudin‐2 [233, 234]. Plexin B2 on the surface of hepatocytes activates class IV semaphorin signaling in tumor cells, promoting their epithelialization mediated by Krüppel‐like factor 4 (KLF4) [235]. Hepatocytes not only release CCL3 and insulin‐like growth factor‐1 to directly regulate the invasiveness [236, 237], but also transport lipid metabolites to provide nutrients for tumor cells [238].

Hepatocytes also play an immunomodulatory role, mainly reflected in attracting myeloid cell infiltration and inducing their functional polarization, as well as inhibiting the cytotoxic immune‐cell function. Under stimulation from liver surgery or CXCL6 derived from metastatic tumor cells, the IL‐6/STAT3/SAA signaling pathway in hepatocytes is activated, promoting MDSCs infiltration, NETs generation, and macrophage M2 polarization [239, 240]. Hepatocyte‐derived fibrinogen‐like protein 1 suppresses CD8+ T‐cell and NK‐cell function through the receptor LAG‐3, thereby facilitating metastasis progression [241]. Additionally, extracellular vesicles released by tumor cells disrupt hepatocyte morphology and function via TGF‐β1/SMAD‐dependent EMT [242]. The inflammatory response at the margin of metastases induces Fas expression in adjacent hepatocytes, making them susceptible to damage by Fas‐ligand‐bearing lymphocytes or tumor cells, thereby promoting tumor invasion [243].

#### 4.1.2 Liver Sinusoidal Endothelial Cells

In addition to their role in extravasation, the continuous interaction between LSECs and metastatic tumor cells is a critical driver of metastatic progression. Research has shown that the transcriptome of LSECs is altered in the presence of metastases, with tumor‐associated LSECs exhibiting greater transcriptomic similarity to venous endothelial cells than to sinusoidal endothelial cells [244]. In vitro coculture experiments have demonstrated that LSECs and metastatic tumor cells interact via ICAM‐1/LFA‐1, promoting the release of tumor‐derived PGE‐2, IL‐6, VEGF, and MMPs, as well as LSEC‐derived IL‐1β, IL‐6, and TNF‐α into the TME [245]. LSEC‐derived LSECs lectin and kallikrein‐related peptide 10 enhance the invasive capacity of metastatic tumor cells and promote angiogenesis [246, 247]. LSECs secrete fibronectin extra domain A, which acts on integrin α9β1 on metastatic tumor cells, activating focal adhesion kinase and Rac signaling to strengthen cytoskeletal polarity [248]. Studies have shown that compared with primary tumors, liver metastatic tumors have a higher proportion of LSECs and more severe LSEC senescence. High expression of KLF4 accelerates LSEC aging, which promotes tumor cell migration through the senescence‐associated secretory phenotype and induces a local immunosuppressive TME [249]. Additionally, LSECs regulate the adhesion of leukocytes and metastatic tumor cells via the cell surface adhesion molecule lymphatic vessel endothelial hyaluronan receptor‐1, influencing the immune killing of metastatic tumor cells [250].

#### 4.1.3 Hepatic Stellate Cells

HSCs within liver metastases can be activated by tumor‐derived signaling molecules such as FGF19 and TGF‐β, as well as by hypoxia or stromal stiffness [251, 252, 253, 254]. Once activated, HSCs contribute to metastasis by enhancing tumor invasiveness, remodeling the extracellular matrix, and modulating immune responses. HSCs infiltrate avascular metastatic lesions before vascular endothelial cells and induce promitotic, antiapoptotic, and promigratory effects in endothelial cells through high expression of VEGF [253]. HSCs also play a role in resistance to antimetastatic therapy. Studies have shown that bevacizumab induces metastatic tumor cells to highly express FGF‐binding protein 1 (FGFBP1), which, in turn, drives the expression of fibroblast activation protein α (FAPα) in HSCs via the FGF2/FGFR1/ERK1/2/EGR1 signaling pathway. HSCs subsequently promote metastatic tumor cell EMT and recruit MDSCs through the CXCL5–CXCR2 axis [255]. Additionally, HSCs are involved in the process of reshaping the immune characteristics of the LMN. HSCs recruit and drive the differentiation of macrophages, DCs, and monocytes while inducing T‐cell proliferation and apoptosis. HSCs also recruit neutrophils, facilitating their infiltration and mediating the formation of NETs within the LMN [251, 256].

#### 4.1.4 Kupffer Cells

The functions of KCs in liver metastasis are dynamically moldable, exhibiting early metastasis‐inhibiting and late metastasis‐promoting effects. In the late stages, KCs often promote angiogenesis while inhibiting lymphocyte infiltration and cytotoxicity [14, 257]. Dectin‐2 is specifically expressed in KCs, but not in bone marrow‐derived or alveolar macrophages, where it mediates the uptake and clearance of metastatic tumor cells [258]. Metastatic tumor cells downregulate the expression of erythroid membrane‐associated protein (ERMAP) to reduce activation of the galectin‐9–Dectin‐2 axis in KCs, thereby evading KC phagocytosis [259]. The pathological characteristics of the liver also influence KC function. Cirrhosis‐associated KCs upregulate the expression of FasR on metastatic tumor cells, making them sensitive to FasR‐mediated apoptosis and preparing them for clearance by lymphocytes, potentially mediated by TNF‐α [260, 261]. Conversely, NAFLD induces KCs to secrete CXCL5, which recruits CXCR2+ MDSCs to support metastatic growth [262]. Additionally, extrahepatic factors, such as the composition of the gut microbiota, may affect KC proliferation, although the underlying molecular mechanisms remain unclear [263].

Some studies have suggested that KCs are a highly promising target for the treatment of liver metastasis. KCs serve as a supplement to macrophages within the LMN and also participate in the regulation of T cell function [264]. In the late stages of metastasis, KCs are often excluded from metastatic lesions. Although they do not directly contact tumor cells, KCs can be activated by exogenous β‐glucans to highly express Irf7 and ISG15, which induce T‐cell‐mediated immune responses through antigen presentation. This activation enhances the sensitivity of metastatic lesions to immunotherapy, thereby inhibiting metastasis progression [265]. Additionally, disrupting the expression of MafB and c‐Maf in KCs using engineered bacteria has been shown to promote KC proliferation and functional reprogramming. Activated KCs regain their phagocytic ability against metastatic tumor cells and enhance antitumor T‐cell responses [266].

#### 4.1.5 Tumor‐Associated Macrophages

TAMs in liver metastasis are a heterogeneous population formed by the infiltration of circulating monocytes, distinct from KCs [267]. Typically, TAMs are classified into two subtypes: M1 and M2. Induced by factors from the LMN and tumor cells, TAMs often lose their tumor‐killing function and polarize toward the M2 phenotype. The activation of PI3K/Akt/PTEN, TGF‐β, and JAK/STAT signaling pathways, or by inhibiting suppressor of cytokine signaling 6 are critical for driving TAM polarization. Several miRNAs (miRNA‐25‐3p, miRNA‐130b‐3p, miRNA‐425‐5p, and miRNA‐106a‐5p) encapsulated in tumor‐derived exosomes, along with IL‐8, CCL2, BMP, and CTHRC1 have been shown to induce this transition [268, 269, 270, 271].

Hepatocytes directly regulate TAM differentiation by releasing vesicles that upregulate the expression of mitogen‐activated protein kinase‐associated protein 1 (MAPKAP1) [272]. They also promote the release of cysteine‐rich angiogenic inducer 61 in metastatic tumor cells through the YAP signaling pathway, thereby remodeling TAM functions [42]. Myofibroblastic fibroblasts are activated in a STAT6‐dependent manner by macrophage‐derived progranulin and tumor cell‐secreted leukemia inhibitory factor, subsequently regulating the TAM phenotype via osteopontin secretion [273]. Efferocytosis by macrophages within the LMN does not maintain tissue homeostasis but instead promotes TAM reprogramming. Mechanistically, progranulin controls lysosomal function, leading to LXRα/RXRα‐mediated functional transformation [274]. Additionally, tissue‐resident microbiota release lactate into the LMN, mediating M2 polarization by suppressing the retinoic acid‐inducible gene 1 (RIG‐I)/mitochondrial antiviral signaling protein/NF‐ĸB signaling pathway [275].

M2 TAMs release vesicles that activate the RAC1/AKT/FOXO1/ATP‐binding cassette transporter A1 signaling pathway, orchestrating the cholesterol metabolism of metastatic tumor cells and influencing metastasis progression [276]. M2 TAMs also regulate lysosomal function and autophagy flux in metastatic tumor cells through glial cell‐derived neurotrophic factor, protecting them from apoptosis under metabolic stress [277]. An essential mechanism by which TAMs facilitate liver metastasis is through the modulation of immune responses. TAMs within the LMN contribute to the formation of an immunosuppressive niche by modulating NK‐cell function and promoting the exhaustion and apoptosis of CD8+ T cells [278, 279].

#### 4.1.6 Neutrophils

Notably, the number of neutrophils in the liver increases dramatically before the appearance of grossly detectable metastatic lesions, highlighting their critical role in preparing for liver metastasis. Metastatic tumor cells primarily upregulate chemokine expression, attracting neutrophils to infiltrate the liver. KIAA1199 is highly expressed in metastatic tumor cells and interacts with TGFBR1/2 to activate the TGF‐β signaling pathway, stimulating the production of CXCL1 and CXCL3 [280]. hnRNPAB enhances CXCL8‐dependent neutrophil recruitment by prolonging the half‐life of MYC mRNA in metastatic tumor cells [199]. The activated Hippo signaling pathway increases glycolytic activity and facilitates neutrophil infiltration mediated by CXCL1 and CXCL5 through the regulation of H3K18 lactylation [281]. Tumor‐derived IL‐1α mediates the polarization of HSCs into inflammatory fibroblasts, which subsequently produce complement C5a and IL‐1β, promoting neutrophil infiltration and the release of NETs [251]. Additionally, macrophage‐derived S100A9 enhances neutrophil infiltration into the metastatic niche [282].

Neutrophils within the LMN exhibit significant heterogeneity and undergo a functional differentiation process, transitioning from antimetastatic to prometastatic phenotypes. Various external factors, including SAA released by hepatocytes in response to ischemia‐reperfusion injury, tumor‐derived cyclic adenosine monophosphate, primary bile acids, and oxidized low‐density lipoprotein, contribute to neutrophil phenotypic polarization [239, 283–285]. In addition to prometastatic mechanisms mediated by NETs, neutrophil‐derived growth arrest‐specific 6 activates the receptor tyrosine kinase AXL on metastatic tumor cells, promoting liver metastasis progression during chemotherapy intervals [286]. Neutrophils secrete anterior gradient‐2 into the extracellular compartment, which increases xCT activity within metastatic tumor cells in a CD98c‐dependent manner, thereby activating the Ras homolog family member A/Rho‐associated protein kinase 2 (RhoA/ROCK2) signaling cascade [287]. Neutrophils also suppress the activation and cytotoxic effects of CD8+ T cells in the LMN through elevated expression of Arg1 and inducible nitric oxide synthase [284].

Although current research highlights the heterogeneity of neutrophil subpopulations in liver metastasis, the mechanisms underlying their phenotypic polarization remain insufficiently explored. The transcription factor Nrf2 is crucial for the formation of neutrophils with an immunosuppressive phenotype. Nrf2 has been found to be responsible for the reprogramming of multiple metabolic pathways of neutrophils within the LMN, including carbon monoxide metabolism, purine metabolism, the TCA cycle and fatty acid metabolism, as well as the suppression of proinflammatory cytokine expression, thereby maintaining their immunosuppressive state [288]. Given their numerical advantage and flexible functional reshaping, targeting the functional transformation of neutrophils is emerging as a promising therapeutic avenue in the fight against liver metastasis.

#### 4.1.7 NK Cells

Previous studies have shown that NK cells induce apoptosis in metastatic tumor cells through the Ca^2+^‐dependent perforin/granzyme pathway or the Fas‐mediated mechanism [289]. However, the cytotoxic function of NK cells is often impaired during metastasis. In circulation, NK‐cell‐mediated elimination of CTCs is downregulated via the HLA‐E:CD94–NKG2A axis [188]. In the metastatic sensing stage, NK cells take up tumor‐derived vesicles, undergo glucometabolic remodeling, and exhibit reduced expression of NKG2D, CD107a, TNF‐α, and IFN‐γ [290]. During the metastatic support stage, NK cells lose their ability to maintain dormancy, a process regulated by HSC‐derived CXCL12 signals [217]. Single‐cell sequencing analysis of primary tumors and liver metastasis samples has revealed that, compared with the primary TME, the number of NK cells is significantly increased in the LMN [129]. However, under the influence of TGF‐β, the cytotoxic NK‐cell subpopulation in the LMN is consistently reduced [291].

Molecules that regulate the functional differentiation of NK cells in liver metastasis have also been explored. Studies suggest that retinoid‐related orphan nuclear receptor alpha (RORα) may serve as an important molecular switch regulating NK‐cell cytotoxic function in the LMN by controlling the expression of various effector molecules. Activation of RORα with agonists has been shown to effectively inhibit liver metastasis progression [292].

#### 4.1.8 T Cells

T cells in the LMN are often in an immunosuppressive state, with impaired antigen presentation and cytotoxic functions, losing the ability to limit metastasis progression. Circulating CD8+ T cells migrate to the liver, where they are influenced by the local niche and fail to become effectively activated, leading to exhaustion and eventual apoptosis [293]. For example, metastatic tumor cells exhibit increased expression of chondroitin sulfate synthase 1 compared with primary tumors. This elevated expression induces CD8+ T‐cell exhaustion through the activation of succinate metabolism and the upregulation of PD‐L1 in tumor cells [294]. Single‐cell sequencing has revealed diverse CD8+ T‐cell subpopulations within the LMN. Understanding the mechanisms behind CD8+ T‐cell dysfunction may uncover key molecular targets to reverse the overall immunosuppressive state.

The generation and expansion of CD4+ T cells depend on signaling molecules derived from the tumor and complex interactions with other immune cells. Studies have shown that the expression of checkpoint genes in metastatic lesions is markedly elevated compared with primary tissues, predominantly within Treg cells [129]. Targeting the depletion or inactivation of Treg cells has been shown to effectively suppress metastasis progression [295, 296].

### 4.2 Spatial Redistribution

**FIGURE 4:**
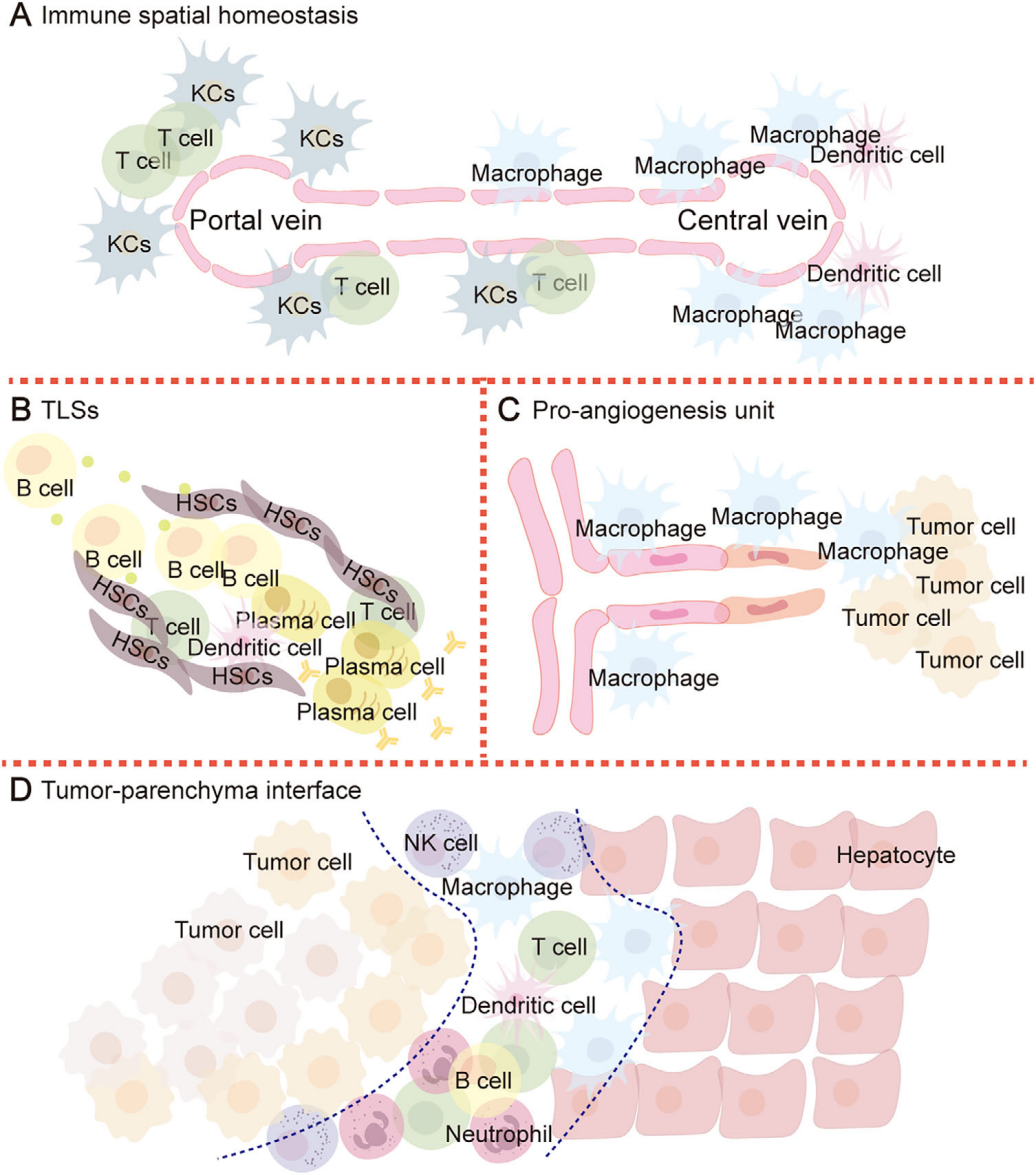
Spatial redistribution of cells involved in liver metastasis. (A) Immune spatial homeostasis: The liver is a highly organized organ with immune cells having inherent distribution tendencies; (B) TILs: TILs form around metastatic lesions, and their number and maturity are associated with patient prognosis; (C) proangiogenesis unit: Macrophages come into contact with endothelial cells locally, inducing the formation of tumor vasculature around liver metastatic lesions. (D) Tumor–parenchyma interface: The interface between liver metastatic tumors and surrounding tissues is infiltrated with a large number of immune cells, and the unique immune characteristics of this zone may significantly affect the biological behavior of metastatic tumors.

Although single‐cell sequencing technology has provided a deeper understanding of the transcriptional characteristics and population heterogeneity of immune cells, this perspective alone sometimes fails to fully explain tumor immune evasion. The liver has inherent immune compartments, with KCs and T cells typically enriched in the portal area, while macrophages and DCs are concentrated in the central area. However, liver metastasis is often accompanied by the spatial redistribution of immune cells, which ultimately alters the global immune response. Two primary spatial characteristics have been extensively explored: the local functional units formed by the aggregation of specific cell populations and the immune status of the tumor–parenchyma interface (Figure 4).

#### 4.2.1 Tertiary Lymphoid Structures

The location and composition of tertiary lymphoid structures (TLSs) within tumors are closely associated with patients’ clinical outcomes [297]. Studies have shown that a high level of tumor‐infiltrating lymphocytes and the presence of TLSs are valuable predictive factors for better overall survival in patients with metastasis [298]. In preclinical models, TLSs have also been linked to favorable responses to immunotherapy. However, the mechanisms underlying TLS formation and their functional characteristics in liver metastases remain unclear. Zhang et al. reported that TLSs located within 2 mm of the border of liver metastases may represent the frontline of antitumor immunity. The distinct distribution and maturation of TLSs could improve the accuracy of predicting clinical prognosis in patients with liver metastases. Mechanistically, a specific inflammatory fibroblast subtype adheres around TLSs, releasing CCL19 to attract CCR7+ B cells, which infiltrate TLSs and support T‐cell survival. In TLS‐positive metastatic tumors, IgG‐positive plasma cells proliferate extensively, releasing large amounts of IgG antibodies that enhance macrophage‐mediated antitumor activity [299].

#### 4.2.2 Proangiogenesis Unit

Angiogenesis is a critical event in tumor progression. The rapid growth of liver metastatic tumors relies on an adequate blood supply, a process that depends on the physical interaction between endothelial cells and macrophages, as well as the transformation of macrophages. The rapid vascularization of liver metastatic tumors involves CD34+ sprouting endothelial cells, allowing the liver vasculature to quickly extend into and around the metastases. Local clusters of F4/80+ TAMs, derived from KCs, stimulate the expression of key angiogenic molecules through their contact with endothelial cells. Additionally, TAMs directly participate in the tumor's vascular mimicry mechanism, providing an alternative perfusion pathway [14, 300].

#### 4.2.3 Tumor–Parenchyma Interface

The tumor–parenchyma interface is critical for the local expansion and distant spread of malignant tumors, characterized by complex microenvironmental changes and distinct tumor cell features [301]. Studies have shown that T‐cell subsets within this zone often exhibit diverse functional characteristics [302]. Immunosuppressive myeloid subpopulations, such as SPP1+ or TREM2+ cells, have been found to possess boundary recruitment effects, which may underlie the restricted invasion of the peripheral stroma [303, 304]. Although many liver metastases exhibit immune cell infiltration at the tumor–parenchyma interface, immune cells are notably rare in this region in replacement GPs. The mechanisms underlying this distinction remain unclear.

In the specialized regions of liver metastases, immune cells often exhibit high expression of Foxp3, VEGFR‐2, and PD‐L1, along with diminished antigen‐presenting capabilities and compromised tumor‐killing functions [305]. Histologically, larger tumors show stronger PD‐L1 expression and an enrichment of CD8+ T cells at the tumor border, whereas CD8+ T cells are more concentrated in the core of smaller metastatic tumors [306]. Studies have shown that a higher CD8+/FoxP3+ ratio in the metastatic core, rather than at the tumor–parenchyma interface, serves as an independent prognostic factor for postoperative survival [307]. TAMs are found closer to tumor cells at the border than at the tumor core. GPNMB+ TAMs are significantly enriched at the tumor–parenchyma interface and contribute to maintaining local immunosuppressive properties [308]. Neutrophils in direct contact with tumor cells undergo lipid metabolism reprogramming, releasing various prometastatic factors and metabolites that promote metastasis progression [285]. Jiang et al. [309] reported that CD93+ monocytes prevent CD8+ T‐cell infiltration into tumors and activation by producing versican and upregulating PD‐L1 expression. Although it remains unclear whether this mechanism is present in liver metastases, these findings suggest that targeting the spatial remodeling of immune cells could be key to enhancing the therapeutic efficacy of treatments for liver tumors.

Upon reaching the liver, metastatic tumor cells initially elicit a robust antitumor immune response during the metastatic sensing and stress stages, after which only a subset of these cells survives and goes into dormancy. However, in the metastatic support stage, the immune landscape of the LMN transitions to a suppressive state, which facilitates metastatic tumor growth and immune evasion. Specifically, myeloid cells, comprising neutrophils and macrophages, rapidly and persistently infiltrate the liver. Under the influence of diverse signals within the LMN, their innate antitumor functions are dampened; instead, they adopt phenotypes that suppress immune responses. Despite the stress‐induced proliferation of resident immune cells, such as DCs and KCs, their antigen‐presenting capabilities remain compromised. They fail to effectively activate antitumor immunity and, instead, release high levels of protumor molecules and immunosuppressive signals, which further support metastasis progression. Lymphocytes that are recruited from the circulation to the liver fail to achieve optimal activation or proliferation. Instead, they experience functional impairments, leading to exhaustion or apoptosis. Beyond the intricate interactions among immune cells, metastatic tumor cells and liver‐intrinsic nonimmune cells also play an active or passive role in the immune remodeling of the LMN. Additionally, different immune cell subpopulations often coexist in tumor, and their phenotypic transition is also continuous. Spatially, metastasis induces the formation of many local functional units, while also altering immune cells’ global distribution characteristics.

It is important to note that while numerous studies have identified key molecules and pathways associated with the immune cell functional reprogramming in primary tumors, their effectiveness within the highly immunosuppressive LMN has yet to be validated. While targeting the immune cell functional reprogramming holds great promise as a therapeutic strategy, a major challenge remains in achieving precise and sustained drug delivery. Furthermore, despite the high degree of similarity, there are intrinsic differences between the immune systems of mice and humans that must be carefully considered when translating preclinical findings into clinical applications [310]. The mechanisms and feasibility of indirectly targeting immune cell functional reprogramming by intervening in nonimmune components are also worth in‐depth exploration.

## 5 Liver Metastases Managements

### 5.1 Surgery

Surgical resection remains the primary curative strategy for liver metastases, while only a minority of patients undergo liver transplantation. Numerous studies have confirmed that surgical resection is a safe and effective option for patients with colorectal cancer or neuroendocrine tumor liver metastases, significantly improving patient prognosis [311, 312]. While small‐scale studies suggest that surgical resection may be a promising treatment option for liver metastases from other primary tumors, including melanoma, esophageal cancer, pancreatic cancer, and adrenocortical carcinoma, high‐quality evidence supporting its efficacy is still lacking [313, 314, 315, 316, 317]. Additionally, the role of liver transplantation in treating patients with liver metastases remains controversial [318, 319].

There is a perspective that liver metastasis signifies systemic tumor invasion, and the trauma of surgery may outweigh its survival benefits for some patients. While successful surgical cytoreduction appears to improve overall survival, the secondary effects of surgery‐including physical dissemination of tumor cells, systemic inflammation, awakening of dormant metastatic tumor cells, and the protumorigenic effects associated with liver regeneration are difficult to control, complicating the assessment of the true benefits of surgery for individual patients [320, 321, 322]. With advancements in multidisciplinary treatment models, the definition of resectability and surgical indications has gradually expanded, though the challenge lies in the rational selection of patients. Some advanced‐stage patients have achieved survival benefits through conversion surgery following neoadjuvant therapy [323, 324].

### 5.2 Ablative Therapy

Ablative therapies are local treatment methods that involve less trauma and fewer complications, effectively inhibiting the growth of local liver tumors while preserving surrounding normal tissues. Compared with systemic therapy alone, combining systemic therapy with radiofrequency ablation ± surgical resection as an active local treatment has provided greater survival benefits for patients with liver metastases [325]. Patients who underwent surgical resection demonstrated longer overall survival and progression‐free survival than those who received radiofrequency ablation, although the incidence of postoperative complications was lower in the radiofrequency ablation group [326, 327]. Additionally, for liver metastases of specific sizes or locations, microwave ablation has been found to be more effective than radiofrequency ablation [328]. A multicenter prospective cohort study showed that microwave ablation achieved therapeutic effects comparable to surgical local resection [329]. Further randomized controlled trials are needed to establish the efficacy, indications, and optimal timing for ablative treatment.

### 5.3 Chemotherapy

Chemotherapy, as the cornerstone of systemic treatment, is used not only for palliative care of unresectable liver metastases but also as neoadjuvant therapy for initially resectable liver metastases, conversion therapy for potentially resectable liver metastases, bridging treatment before liver transplantation, and adjuvant therapy for high‐risk recurrent liver metastases. While chemotherapy primarily targets tumors prone to widespread dissemination or those already metastasized, it rarely cures or eradicates liver metastases. Furthermore, there are currently no effective regimens specifically targeting dormant metastatic tumor cells or micrometastases. As a result, excessive chemotherapy often fails to prevent metastasis and instead leads to systemic toxicity.

In preclinical models, chemotherapy has been found to accelerate the process of metastasis. Chemotherapy induces increased levels of inflammation‐related factors in local liver tissue, activates mesenchymal stem cells and endothelial cells, and attracts myeloid cells to infiltrate the TME [286, 330, 331]. Additionally, chemotherapy enhances the prometastatic abilities of platelets and LSECs and directly increases the invasive capabilities of metastatic tumor cells [332, 333, 334]. However, the relationship between these findings and their clinical relevance requires further exploration.

### 5.4 Radiotherapy

Radiotherapy is a noninvasive local treatment that has been demonstrated to be safe, with a low risk of transient biochemical liver toxicity in the treatment of liver metastases [335]. An international patient registry survey reported that stereotactic body radiotherapy for liver metastases achieves good local control rates and overall survival, which are influenced by the dose and tumor volume. Overall survival varies depending on the primary tumor type [336]. In patients with metastatic lung cancer, radiotherapy improves progression‐free survival and effectively modulates the immune therapy response of tumors with low PD‐L1 expression [337]. The role of radiotherapy in multidisciplinary treatments for liver metastases from different primary sites warrants further prospective studies. Additionally, some studies have found that radiotherapy can accelerate metastasis progression in preclinical models, although the underlying mechanisms remain unclear [338].

### 5.5 Targeted Therapy

Targeted therapy is an ideal strategy for precision cancer treatment, minimizing toxic side effects on normal tissues. Currently, drugs such as bevacizumab (targeting VEGF), cetuximab (targeting EGFR), and multitarget agents such as regorafenib are used in combination with first‐line chemotherapy, significantly improving the objective response rate and conversion treatment success rate in patients with liver metastases [339, 340, 341]. In recent years, new targets (such as NTRK fusions, FGFR2 fusions, KRAS G12C mutations, and Claudin18.2) and new technologies (such as antibody‐drug conjugate, proteolysis targeting chimera and bispecific antibody) are expected to further improve the safety and efficacy of targeted therapy.

### 5.6 Immunotherapy

Immune evasion enables tumors to escape destruction by the host's immune system, resulting in unchecked proliferation. Increasing evidence suggests that compared with the primary TME, the LMN exhibits higher levels of immunosuppression [129, 342, 343]. Patients with liver metastases derive less benefit from immunotherapy compared with those without liver metastases. Liver metastases affect immunotherapy efficacy by altering the body's antitumor response [344, 345]. The metastatic liver attracts activated CD8+ T cells from the circulation and promotes their apoptosis [17]. Additionally, inducing T‐cell dysfunction is a key mechanism underlying the systemic immune tolerance driven by liver metastases [346].

Restoring host immune functions or providing additional effective immune components has emerged as a promising approach in cancer treatment; however, it must be acknowledged that this is extremely challenging in clinical practice. Preclinical studies have found that systemic depletion of certain cell types does not achieve stable antitumor effects [347], and such methods do not meet the clinical ethical requirements. The primary therapeutic strategy targeting immune cell function is the application of immune checkpoint inhibitors (ICIs), which specifically break the suppressive state of immune cells. However, ns cannot reverse the immune features of the TME. ICIs either alone or in combination with traditional treatments, benefit only a subset of patients with liver metastases [348, 349, 350]. A higher mutational burden and the production of immunogenic neoantigens partially explain the sensitivity of some patients to these therapies [351]. Studies have confirmed that ICIs cause immune‐mediated pathology in various organs, including the gastrointestinal tract, lungs, and others; these issues limit their clinical application [352, 353].

Adoptive cell therapy is an emerging immunotherapy strategy that utilizes or modifies the body's immune cells to eliminate tumors. Small‐sample studies have shown that CAR‐T cell therapy, TCR‐T cell therapy, and cytotoxic lymphocyte therapy for liver metastases are tolerable and promising [354, 355, 356]. Preclinical studies are also exploring more direct or adjunctive strategies to improve the efficacy of adoptive cell therapy [357, 358]. In recent years, CAR‐NK cells and CAR‐macrophages have been introduced into the treatment of solid tumors, offering a supplement or alternative to CAR‐T cell therapy [359]. Additionally, the identification of tumor neoantigens and the development of tumor vaccines have demonstrated significant therapeutic potential for tumor metastases [360]. However, adoptive cell therapeutic strategies seem to be unable to effectively break through the immunosuppressive barriers of solid liver metastatic tumors and also face technical challenges in ex vivo cell preparation and expansion [361].

## 6 Ongoing Preclinical and Clinical Explorations

Currently, many preclinical and clinical studies focus on liver metastasis treatment by targeting metastatic tumor cells, stimulating the systemic immune response, dismantling the physical barriers of tumors, and modulating the immune cell functional differentiation in vivo, as summarized in Table 3. Additionally, the challenge of how to accurately select and synergistically combine these therapeutic strategies, especially in light of individual heterogeneity, is a critical issue for future research [362].

**TABLE 3:** Summary of current potential targets and mechanisms for liver metastasis treatment in preclinical animal experiments and clinical trials.

Type	Reagent	Target	Mechanisms	Phase	References
Regulate tumor immunity	HCW9218	TGF‐β; IL‐15	Remodels immunosuppressive TME; enhances immune cell infiltration and cytotoxicity	Phase 1b/2	NCT05304936
	Galunisertib	TGF‐β	Inhibits canonical TGF‐β pathway	Phase 1	NCT02734160
	M7824	TGF‐β; PD‐L1	Activates the innate and adaptive immune systems	Phase 1	NCT02517398
	BCA101	EGFR; TGFβ	Promotes the production of proinflammatory cytokines; suppresses VEGF secretion; regulates CD4+ T‐cell differentiation	Phase 1	NCT04429542
	SHR‐1701	PD‐L1; TGF‐βRII	Regulates tumor immunity	Phase 1	NCT03710265
	AZ13381758	CXCR2	Enhances T cell infiltration and confers sensitivity to anti‐PD1 therapy	Preclinical phase	[347]
	PF‐04136309	CCR2	Restores antitumor immunity	Phase 1	NCT01413022
	Sargramostim	GM‐CSF	Regulates innate and adaptive immune responses	Phase 2	NCT00003125
	PEG	IL‐10	Regulates MHC class II antigens and immune response	Phase 3	NCT02923921
	αPD‐L2	PD‐L2	Selective activates CD8+ T‐cell response	Preclinical phase	[296]
	Q702	AXL; MerTK; CSF1R	Regulates non‐T cell immune response	Phase 1b/2	NCT05438420
	GWN323	GITR	Attenuates T reg‐mediated suppression; enhances tumor‐killing by effector T cells	Phase I/Ib	NCT02740270
	CFTRinh172 (CFTRi)	CFTR	Impairs macrophage conversion; regulates CD8+ T‐cell functions	Preclinical phase	[274]
	Mitazalimab	CD40	Activates antigen‐presenting cells; improves expansion of antigen‐specific T cells	Phase 2	NCT04888312
	CDX‐1140	CD40	Activates DCs and B cells	Phase 1	NCT03329950
	BATs		Activates antitumor immune response	Phase 2	NCT02620865
	NM/PPcDG/D		Inhibits MDSC recruitment and functions	Preclinical phase	[363]
Target nonimmune component	SLM	HIFs	Enhances prolyl hydroxylase‐dependent HIF degradation; stabilizes tumor vasculature; downregulates of oncogenic miRNAs	Phase 1/2	NCT05363631
	Elraglusib	GSK3	Inhibits tumor growth and T‐cell functions	Phase 2	NCT05077800
	Pirfenidone	Fibrosis	Reduces collagen and hyaluronan levels; increases blood vessel functionality and perfusion	Phase 1/1b	NCT03177291
	CCT365623	EGFR	Inhibits metastatic tumor growth	Preclinical phase	[364]
	CHKI‐03	CHKA	Inhibits metastatic tumor growth	Preclinical phase	[365]
	Ephrin B2 Fc chimaera protein	VEGFR	Induces vascular normalization and inhibits CTC cluster formation	Preclinical phase	[366]
	cGAS‐STING	STING	Inhibits the reactivation of dormant metastasis	Preclinical phase	[225]
	Anti‐mouse IL‐6R Antibody (15A7)	IL‐6	Inhibits stromal cell IL‐6 signaling	Preclinical phase	[156]

Abbreviations: CFTR, cystic fibrosis transmembrane conductance regulator; CHKA, choline kinase alpha.; GITR, glucocorticoid‐induced tumor necrosis factor receptor; GSK3, glycogen synthase kinase‐3. GITR, glucocorticoid‐induced tumor necrosis factor receptor; CFTR, cystic fibrosis transmembrane conductance regulator; GSK3, glycogen synthase kinase‐3; CHKA, choline kinase alpha.

## 7 Conclusion and Prospects

Liver metastasis is a complex, multistep process involving not only the active plasticity of metastatic tumor cells but also dynamic changes within the TME, as well as their intricate interactions, all of which significantly influence the final outcome. Currently, the volume of liver metastasis research is rapidly increasing, with numerous key molecular targets being identified, showing promising prospects for the development of tailored therapeutic strategies. Given the high plasticity of metastatic tumor cells, intervention strategies based on these findings must consider the specific stage of liver metastasis. For example, critical regulatory molecules or epigenetic modifications that function in the primary tumor may not function in the same mechanism or be preserved during tumor cell dissemination, metastatic tumor dormancy, and ectopic tumor growth. Additionally, there is also a substantial biological disparity between micrometastases and macrometastases, further complicating the therapeutic options. The complex interactions between cells during liver metastatic are dynamic, with both resident and recruited cells playing dual roles. A delicate balance among these roles ultimately dictates whether metastatic tumor cells will colonize, enter a state of dormancy, proliferate, or ultimately die. Hence, precisely targeting specific cellular interactions presents a significant challenge. It is worth noting that at the spatial level, enhancing the infiltration quality of cytotoxic immune cells within the LMN, reprogramming the immunosuppressive characteristics of the tumor–parenchyma interface, and inhibiting the formation of prometastatic local functional units are promising adjunctive therapeutic strategies.

Currently, numerous factors hinder the progress of research on the molecular mechanisms and clinical management of liver metastasis. The significantly faster rate of cancer evolution in mice compared with humans, along with interspecies differences in their immune systems, and the prevailing research approach that relies on preventing or reducing tumor growth to evaluate therapeutic efficacy—all these factors necessitate careful consideration when translating preclinical research findings [146]. Exploring mechanisms of drug resistance represents a critical tumor research direction. However, overcoming drug resistance in metastatic tumors seems more challenging. Metastatic tumor cells possess enhanced adaptive capabilities, which further augment their resistance to cytotoxic or targeted therapies [141]. In addition, the lack of effective biomarkers to predict metastasis propensity and early detection of tumor metastasis often leads to delays in diagnosis and treatment.

Future liver metastasis management should fully consider individual heterogeneity, and with the aid of molecular detection and imaging technologies, identify the stages of liver metastasis to implement targeted interventions. Although the advancements in multidisciplinary treatment models have improved the survival time of patients with liver metastasis, it is regrettable that only a small proportion of the population benefit from it. For the majority of tumor patients, current delays in diagnosis and intervention often lead to relentless progression of metastasis. Therefore, it is also imperative to explore palliative therapeutic strategies to improve patients’ quality of life and alleviate symptoms.

## Author Contributions

Wenchao Xu and Jia Xu contributed equally to this review article. Wenchao Xu and Jia Xu drafted the manuscript and prepared the figures. Jianzhou Liu, Nanzhou Wang, and Li Zhou helped in collecting the related literatures and participated in the discussion. Junchao Guo designed the review and revised the manuscript. All authors have read and approved the final manuscript.

## Ethics Statement

Not applicable.

## Conflicts of Interest

The authors declare no conflicts of interest.

## Data Availability

Not applicable.
